# Gestation Within a Non-communicating Rudimentary Horn of a Unicornuate Uterus: A Case Report

**DOI:** 10.7759/cureus.92641

**Published:** 2025-09-18

**Authors:** Bodoor Saad S Alshareef, Abdulmalik K Alaqeeli, Ehab Wahbah, Yousef Albayati, Yousef Shata, Mohammed Ibrahim Alsabti, Inas Babic

**Affiliations:** 1 Obstetrics and Gynecology, Prince Sultan Military Medical City, Riyadh, SAU; 2 College of Medicine, Alfaisal University, Riyadh, SAU; 3 Radiology, Prince Sultan Military Medical City, Riyadh, SAU

**Keywords:** congenital uterine malformation, early pregnancy ultrasound, first trimester, missed abortion, müllerian ducts, unicornuate uterus

## Abstract

Pregnancy in a non-communicating rudimentary horn of a unicornuate uterus is a rare and potentially dangerous condition due to the risk of rupture and maternal morbidity. In this report, we discuss the case of a 39-year-old woman with three previous term deliveries who was diagnosed in the first trimester with a rudimentary horn pregnancy. She remained asymptomatic and underwent successful surgical excision of the horn containing the pregnancy. This case highlights the importance of early recognition and timely surgical management in preventing life-threatening complications.

## Introduction

Congenital uterine malformations affect the general population at a rate of approximately 1-10% [1]. Müllerian duct anomalies are congenital uterine malformations that are caused by abnormal development, fusion, or absorption of the Müllerian ducts [2]. 

A unicornuate uterus is a type of Müllerian duct anomaly that is caused by the failure or underdevelopment of one of the Müllerian ducts, producing an asymmetric, hemicavitary uterus with an elongated, curved configuration [2,3]. It affects 0.1% of the general population, and 84% of unicornuate uteri are associated with a contralateral rudimentary horn [3]. This condition may be characterised by the presence of an underdeveloped or rudimentary horn, as well as communication between the uterus and an endometrium-lined cavity. Pregnancy in a rudimentary horn carries a high risk of rupture, typically between 10-15 weeks of gestation [4], which may cause life-threatening hemoperitoneum and contribute to a reported maternal mortality rate of 6-23% [5].

The American Fertility Society distinguishes four subtypes of unicornuate uterus: rudimentary horn with a cavity communicating with a unicornuate uterus, with a non-communicating cavity, without a cavity, and a rudimentary horn [6]. The most common subtype of unicornuate uterus is a non-communicating rudimentary horn [7]. Because many patients are asymptomatic, the diagnosis of this anomaly is usually delayed [8].

We present a rare case of a non-communicating rudimentary horn pregnancy in a unicornuate uterus, diagnosed after three prior full-term deliveries.

## Case presentation

A 39-year-old woman, gravida 4 para 3, presented at 12 weeks of gestation for a routine early pregnancy ultrasound. She was asymptomatic at the time of presentation, with no abdominal pain, vaginal bleeding, or systemic complaints. Her obstetric history included three previous full-term deliveries, one of which was by cesarean section 10 years earlier for a non-reassuring cardiotocography. She had no significant medical or surgical history otherwise.

**Figure 1:**
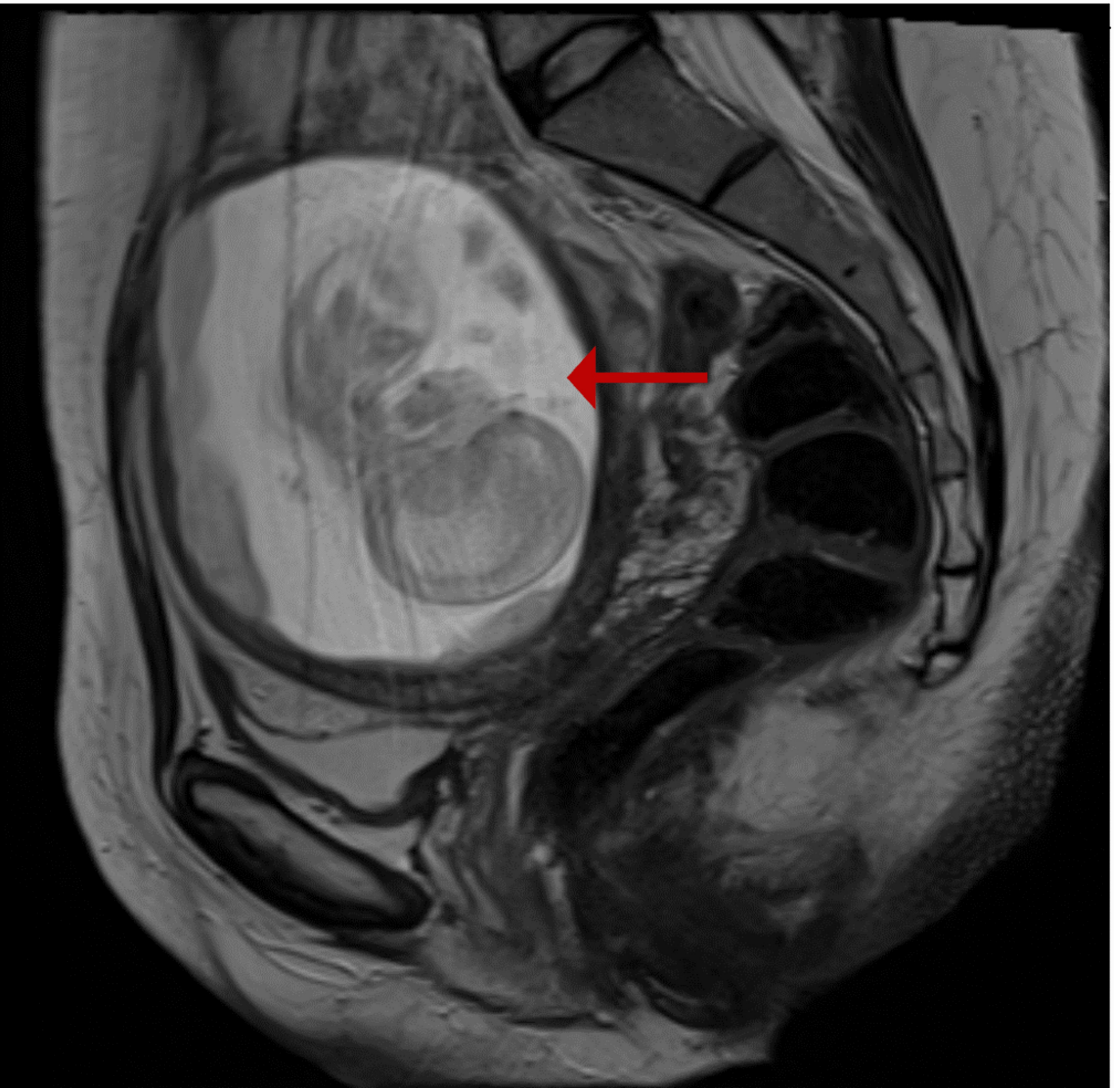
Sagittal section of MRI pelvis showing rudimentary horn containing fetus (red arrow).

**Figure 2:**
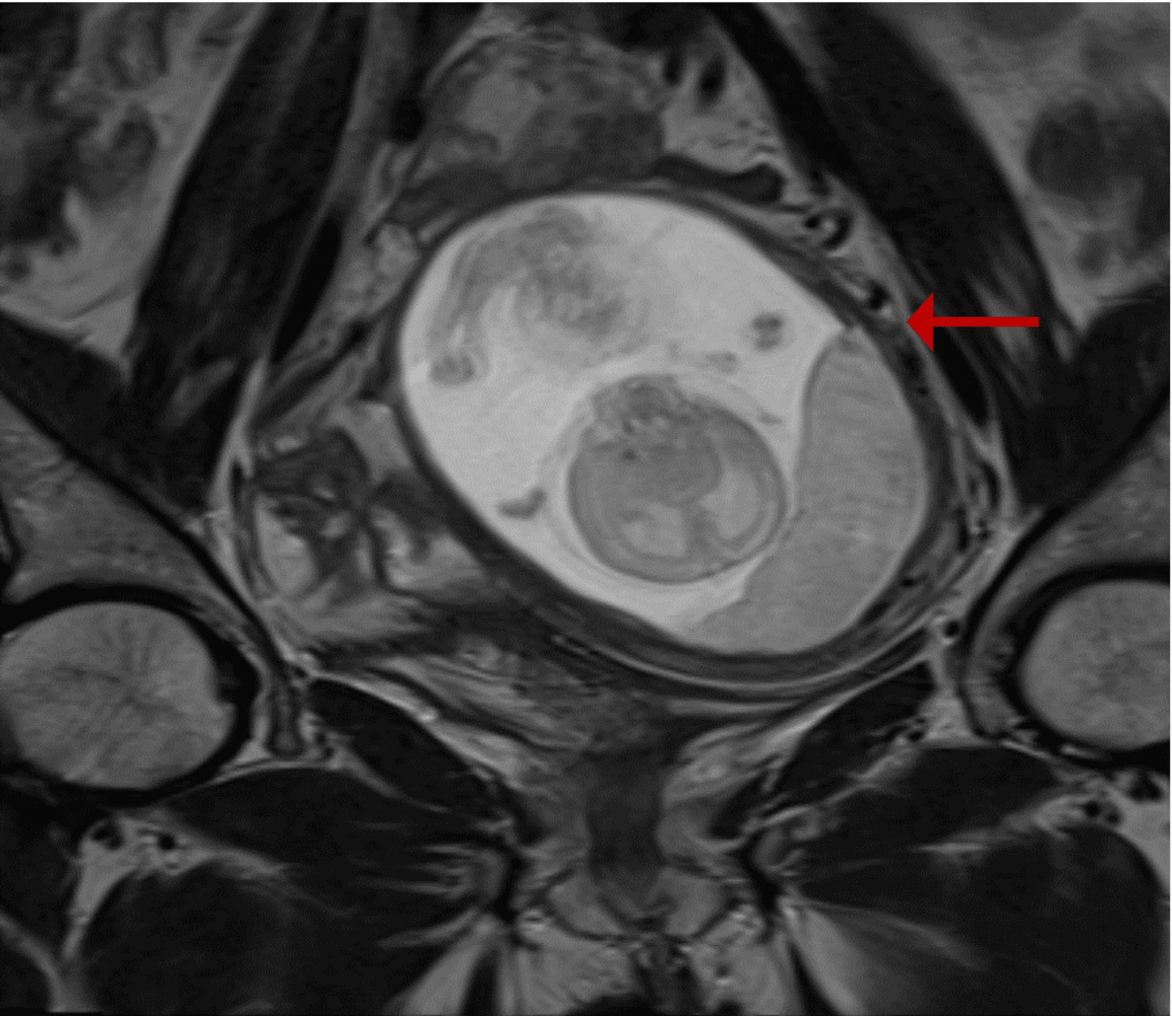
Focused coronal section of MRI pelvis showing the rudimentary horn with fetus and placenta (red arrow).

**Figure 3:**
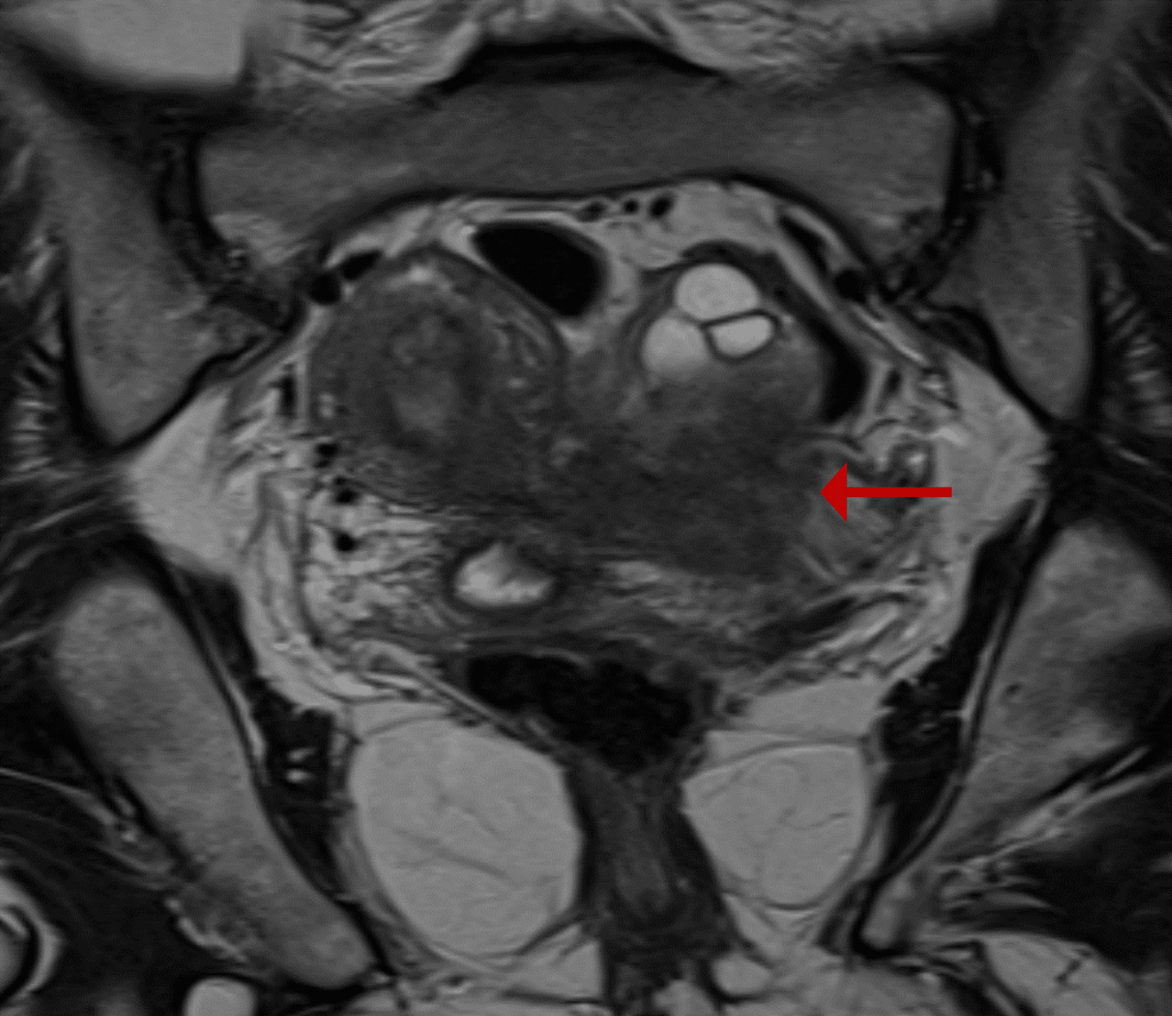
T2 weighted coronal section of MRI pelvis showing rudimentary horn on the left side of the uterus (red arrow).

A pelvic ultrasound performed independently by two physicians suggested an abnormally located pregnancy. Subsequent magnetic resonance imaging (MRI) of the pelvis (Müllerian protocol) demonstrated a right-sided unicornuate uterus with preserved zonal anatomy and a left-sided rudimentary horn containing a gestational sac (Figures 1, 2). A hyperintense linear tract on T2-weighted images initially raised suspicion of possible communication (Figure 3).

The patient was admitted for medical management of a missed abortion and received three courses of misoprostol without successful evacuation. Examination under anesthesia with hysteroscopy revealed a single cervical os with no access to the gestational sac, confirming a non-communicating rudimentary horn.

**Figure 4:**
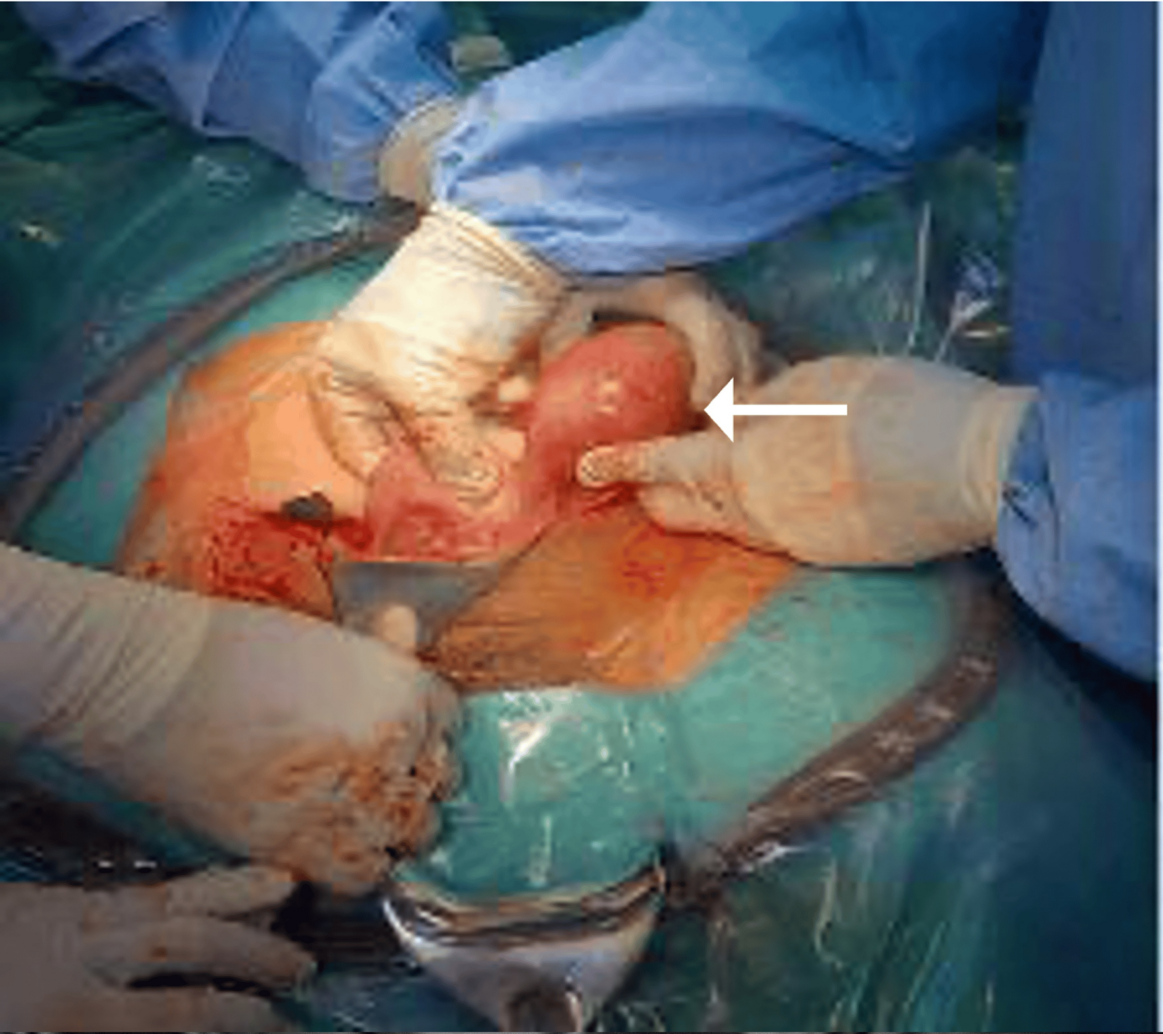
Intraoperative macroscopic image of uterus with non-communicating horn (white arrow).

Given these findings, the patient underwent laparotomy with excision of the left rudimentary horn and the contained pregnancy. Intraoperative findings included a pregnancy localized within the rudimentary horn, a normal right fallopian tube and ovary, and an abnormal left tube attached to the cervix. There was no demonstrable communication between the horns (Figure 4). Estimated blood loss was 100 mL, and there were no intraoperative complications.

Histopathological examination of the excised specimen confirmed chorionic villi within uterine-type tissue, consistent with pregnancy in the rudimentary horn. The patient’s postoperative course was uneventful, and she was discharged in stable condition on prophylactic antibiotics and thromboprophylaxis. At the one-month follow-up, she was asymptomatic, her surgical site had healed well, and she was commenced on contraception, with further imaging follow-up planned.

## Discussion

Müllerian anomalies, where up to 4% of women are affected, include the absence of a uterus, the formation of a half or double uterus, and the formation of a uterus divided by a septum [9].

A unicornuate uterus represents an uncommon congenital malformation caused by incomplete development of one of the Müllerian ducts. According to the American Fertility Society, it can be divided into four categories depending on whether a rudimentary horn exists, if it communicates with the main uterine cavity, and whether it contains functional endometrium [10]. This condition carries clinical importance due to its strong association with reproductive challenges, including infertility, recurrent miscarriage, and obstetric complications such as ectopic gestation and preterm birth [11].

This anomaly accounts for an estimated 2.4-13.7% of all congenital uterine abnormalities. In the majority of cases (about 84%), a rudimentary horn develops on the opposite side, and in 75-90% of these instances the horn is non-communicating [12]. Our case was notable in that the patient presented asymptomatically at 12 weeks of pregnancy. Conception within a non-communicating horn is exceptionally uncommon, occurring in roughly one in 100,000 pregnancies, usually through transperitoneal passage of sperm [13]. Although early pregnancy may remain silent, 80-90% of women experience acute complications in the second trimester, most commonly rupture of the horn, which can lead to massive intra-abdominal bleeding, shock, and even maternal death [14].

Diagnosis of pregnancy in a rudimentary horn during the first trimester is challenging. Although preoperative diagnoses are being reported with increasing frequency, they are typically made in 6-13 weeks of gestation, making early recognition in the first trimester critical. In our case, the pregnancy was successfully diagnosed during the first trimester. Beyond this period, detection of a rudimentary horn pregnancy becomes extremely difficult. Undiagnosed rudimentary horn pregnancies invariably lead to rupture, which occurs in 80-90% of cases between 10 and 20 weeks of gestation [15,16].

Ultrasound has a diagnostic sensitivity of about 26%, and only 14% of cases can be diagnosed before symptoms appear. MRI and hysteroscopy are critical for confirming the diagnosis of a unicornuate uterus with a rudimentary horn, particularly when ultrasound findings are inconclusive [17]. In a recently published case, the diagnosis could not be confirmed prior to using MRI [18]. In our case, diagnostic hysteroscopy was used to reach the final diagnosis, which confirmed that the left rudimentary horn was non-communicating. The mainstay treatment is surgical removal of the pregnancy alongside the rudimentary horn and ipsilateral tube. 

Early recognition of a unicornuate uterus is crucial in order to provide tailored prenatal surveillance and care. Consistent antenatal follow-up enables early identification of complications related to this anomaly and facilitates timely intervention [19]. In our case, the patient did not engage in antenatal care until admission, highlighting the critical need to ensure universal access to comprehensive prenatal services.

Counseling also plays a vital role in managing pregnancies complicated by a unicornuate uterus. Women should be made aware of the heightened likelihood of adverse obstetric outcomes and advised on the benefits of appropriate birth spacing and reliable contraception to optimize reproductive health and minimize risks in future pregnancies [20]. Current recommendations further emphasize delaying conception for at least six months and employing highly effective contraceptive methods during this period.

## Conclusions

A unicornuate uterus containing a non-communicating rudimentary horn is an uncommon congenital anomaly that can have serious consequences for a woman’s reproductive health. It has been linked to complications such as recurrent miscarriage, malpresentation, fetal growth restriction, preterm delivery, and, most dangerously, rupture of the rudimentary horn with the potential for life-threatening hemorrhage. Recognising and diagnosing this anomaly as early as possible-ideally during the first trimester-can greatly reduce maternal risks. The ability to diagnose and classify these conditions quickly and accurately is critical for managing and counselling affected individuals. Advances in diagnostic techniques are required to improve our understanding of these conditions and provide timely intervention.
